# Quantitative Phenotyping of *Xenopus* Embryonic Heart Pathophysiology Using Hemoglobin Contrast Subtraction Angiography to Screen Human Cardiomyopathies

**DOI:** 10.3389/fphys.2019.01197

**Published:** 2019-09-20

**Authors:** Engin Deniz, Stephan Jonas, Mustafa K. Khokha, Michael A. Choma

**Affiliations:** ^1^Department of Pediatrics, Yale University, New Haven, CT, United States; ^2^Department of Informatics, Technical University of Munich, Munich, Germany; ^3^Department of Genetics, Yale University, New Haven, CT, United States; ^4^Department of Diagnostic Radiology, Yale University, New Haven, CT, United States; ^5^Department of Biomedical Engineering, Yale University, New Haven, CT, United States; ^6^Department of Applied Physics, Yale University, New Haven, CT, United States

**Keywords:** Xenopus tadpole, hemoglobin subtraction angiography, human cardiomyopathy, animal model cardiovascular system, videomicroscopy

## Abstract

Congenital heart disease (CHD) is a significant cause of mortality in infants and adults. Currently human genomic analysis has identified a number of candidate genes in these patients. These genes span diverse categories of gene function suggesting that despite the similarity in cardiac lesion, the underlying pathophysiology may be different. In fact, patients with similar CHDs can have drastically different outcomes, including a dramatic decrease in myocardial function. To test these human candidate genes for their impact on myocardial function, we need efficient animals models of disease. For this purpose, we paired *Xenopus tropicalis* with our microangiography technique, hemoglobin contrast subtraction angiography (HCSA). To demonstrate the gene-teratogen-physiology relationship, we modeled human cardiomyopathy in tadpoles. First we depleted the sarcomeric protein myosin heavy chain 6 (myh6) expression using morpholino oligos. Next, we exposed developing embryos to the teratogen ethanol and in both conditions showed varying degrees of cardiac dysfunction. Our results demonstrate that HCSA can distinguish biomechanical phenotypes in the context of gene dysfunction or teratogen. This approach can be used to screen numerous candidate CHD genes or suspected teratogens for their effect on cardiac function.

## Introduction

Congenital heart disease (CHD) occurs in ~8 out of 1,000 live births and effects 1.3 million newborns per year worldwide (Marelli et al., 2007; van der Linde et al., 2011). Currently the prevalence of adults with CHD exceeds the pediatric population, shifting the burden of disease to adulthood, and enlarging the cohort of CHD patients. Despite the early corrective measures and close, multidisciplinary care, adults with CHD still have a significantly higher mortality rate (Pierpont et al., 2007). Sudden death and early onset congestive heart failure remains the most common causes of mortality suggesting that premature myocardial failure may be associated with adult CHD (Sabatine et al., 2006; O'Donnell and Nabel, 2011; Kathiresan and Srivastava, 2012). Currently very little is known about the interactions between the malformed heart and biomechanical function which ultimately affects life expectancy and the quality of life.

Human genomics technologies are now enabling genetic analyses of patients with CHD (Fakhro et al., 2011; Sanders et al., 2012; Zaidi and Brueckner, 2017; Pierpont et al., 2018). Based on the diversity of the genes identified in similar phenotypic patients, we suggest that genetic heterogeneity may explain the differences seen in patient outcomes. To better understand this relationship between a candidate gene and the biomechanical phenotype, we need to develop efficient animal models to evaluate the effect of candidate CHD genes on cardiac function. This would be the first step to investigate the functional variability seen in CHD patients.

Imaging small animal cardiovascular system remains to be a challenge because of the small heart sizes as well as the fast heart rates requiring high resolution, high speed imaging. Cardiac MRI with gating has been used for imaging anesthetized mice and rat cardiovascular system yet image acquisition time reaching to hours making it difficult to use for any screening purposes (Ramirez and Bankson, 2007; Ramirez et al., 2007; Esparza-Coss et al., 2008). Alternatively micro-CT systems are designed to provide fast (1–2 s) imaging. Combined with the cardio-respiratory gating and laborious segmentation, 4D data sets can be formed to measure cardiac function (Yushkevich et al., 2006; Clark et al., 2013). Both of these modalities have limited utilization for rapid, high-throughput screening since they require extensive image processing. As an alternative modality our group and the others have implemented Optical Coherence Tomography (OCT) imaging for small animal cardiovascular imaging (Tearney et al., 1996; Luo et al., 2006; Männer et al., 2009; Yoo et al., 2011; Wang et al., 2016; Deniz et al., 2017; Grishina et al., 2017). Principally OCT imaging is similar to the ultrasound but light is utilized instead of sound and provides cross-sectional images. With scan rates of ~100 2D frames/s and implemented Doppler technology, OCT imaging has been evolving in functional analysis of cardiovascular system in small animal models (Davis et al., 2008; Jenkins et al., 2010). The main disadvantages of OCT are the expense of the commercially available products and the limitations of Doppler application. For accurate quantification of blood flow by doppler application requires high speed systems to prevent phase-wrapping artifacts and also relies on laminar flow. This is very difficult to obtain from motile, trabeculated embryonic ventricle, limiting Doppler OCT application at its current state for accurately quantifying cardiac ventricle function.

We previously demonstrated that hemoglobin contrast subtraction angiography (HCSA) can exploit the wavelength-sensitive absorption of hemoglobin as a molecular-specific source of endogenous flow contrast and can be applied to *Xenopus* tadpole hearts in a straightforward manner (Deniz et al., 2012) using an EOS camera. HCSA can quantify changes in the embryonic cardiac function after perturbation with a calcium channel blocker. In this brief report, we demonstrate that *Xenopus* can be a fast gene-teratogen-function assay and when coupled with HCSA, can be used to quantify physiological cardiac phenotypes over a short period of time. *Xenopus*, as a model of cardiac development, has a favorable balance between human modeling and cost/efficiency that is required for high throughput screening (Warkman and Krieg, 2007; Khokha, 2012). The tadpole cardiovascular system develops within 72 h and remains optically accessible throughout the early stages of development. Importantly, during this period of development, tadpoles can survive with severely malformed hearts since nutrient and O_2_ delivery is not dependent on blood circulation. Here, we capitalized on these advantages to model human cardiomyopathy in *Xenopus* in two ways, first by reducing the expression of the sarcomeric protein myosin heavy chain 6 (MYH6) using morpholino oligonucleotides (MO) and, in a separate set of experiments, by exposing them to the known teratogen ethanol (EtOH).

Cardiac muscle myosin consists of two heavy chain subunits, two light chain subunits, and two regulatory subunits. We used MYH6 morpholino to block the translation start site of the mRNA encoding the alpha heavy chain. It has been shown that mutations in MYH6 gene cause cardiomyopathy both in humans (Hershberger et al., 2010) and *Xenopus* suggesting a well-conserved disease mechanism (Abu-Daya et al., 2009). One substantial advantage of MOs is that gene dosage can be titrated based on the amount of MO injected providing fine dosage control. This is important since most cardiomyopathy patients are heterozygous (Zaidi and Brueckner, 2017) and therefore gene dosage may play a critical role.

In parallel to the knockdown approach, we exposed the developing embryo to EtOH, a known cardiac teratogen, affecting one in eight pregnant women (Floyd and Sidhu, 2004) and leading to fetal alcohol syndrome (FAS). Worldwide FAS affects many children with a prevalence of 23 per 1,000 (Roozen et al., 2016). Patients with FAS also present with structural birth defects including cardiac defects (Jones et al., 2010). To investigate the effects of EtOH on the developing cardiovascular system, studies in zebrafish found increased heart volumes, decreased thickness of the ventricular wall, and decreased basal heart rates (Dlugos and Rabin, 2010). Similarly, in *Xenopus* embryos exposed to EtOH, a reduction in trabeculae formation was found (Yelin et al., 2007). More elaborate work in chick suggested that ethanol-induced alterations in early cardiac function may potentially lead to late-stage valve and septal defects (Karunamuni et al., 2014). Together, EtOH appears to alter myocardial formation and pump function.

In this report we show that HCSA imaging can detect changes in embryonic cardiac function using several different anatomic and physiological metrics, establishing a rapid, non-destructive, low-cost method to investigate cardiac gene-function relationship.

## Materials and Methods

### Xenopus Husbandry

*Xenopus tropicalis* were housed and cared for in our aquatics facility according to established protocols that were approved by the Yale Institutional Animal Care and Use Committee (IACUC) in accordance with NIH guidelines. Female and male mature *Xenopus tropicalis* were obtained from National Xenopus Resource.

### MYH6 Knockdown

*In vitro* fertilization and microinjections were done as previously described (del Viso and Khokha, 2012). In order to deplete *myh6*, we injected fertilized eggs with either 1 or 2 ng of MYH6-MOs and the tracer dye Alexa488 (Invitrogen) at one cell stage. Controls were injected with only tracer dye Alexa488. The advantage of MOs is that “gene dosage” can be titrated based on amount of MO injected providing fine dosage control. We used a translational-blocking start site morpholino oligo (MO) (Morcos, 2007) to deplete the alpha heavy chain, MYH6 (sequence 5′ AGTCTGCCATCAGGGCATCACCCAT-3′–Gene-Tools, LLC) which was previously utilized and verified in *Xenopus* (Abu-Daya et al., 2009). Embryos were injected at the one cell stage with borosilicate glass needles. Post-injections, embryos were incubated in 1/9 Modified Ringer's solution supplemented with 50 μg/ml of gentamycin at 25°C. Injections were confirmed by fluorescent lineage tracing with a Zeiss Lumar fluorescence stereomicroscope at stage 28 and tadpoles further raised at until stage 45 (Nieuwkoop—Faber staging. ~ post-fertilization day 4 at 25°C Nieuwkoop and Faber, 1994) for imaging.

### EtOH Treatment

We incubated *Xenopus* embryos in 1% EtOH in 1/9 Modified Ringer's solution at 25°C following mid-blastula stage (stage 9, Nieuwkoop—Faber staging Nieuwkoop and Faber, 1994) as described previously (Yelin et al., 2007) until stage 45 to determine the effects on the myocardium using HCSA imaging. EtOH solution refreshed daily for 4–5 days until tadpoles reached stage 45.

### Imaging and Image Processing

We used a Nikon SMZ800 stereomicroscope equipped with Canon EOS 5D Mark II digital camera and Sugar CUBE—LED (4,000 Lumens) with ring illuminator. Image acquisition setup: Zoom 6.2/Gain: 0/Depth 14 bit/LED 4,000 Lumens. Fifty RAW images acquired with 0.2 s intervals. High Speed color movie obtained with Motion Xtra N4-IDT, Inc. N4 Camera. For cardiac functional analysis, we embedded Stage 44–45 (post fertilization day 3 at 28°C) tadpoles in low melt agarose and positioned ventral surface exposed to the imaging plane (Supplemental Figure 1A). First, we obtained a scout image to record the tadpole stage, tadpole position and overall development. Next, we imaged the cardiac sac and confirmed that ventricle borders were clearly delineated. We obtained 50 still images (14-bit frames in RAW format) in the time lapse mode with a 0.2 s interframe interval using a Canon EOS 5D Mark II digital camera (Supplemental Figure 1B). Acquiring 50 images effectively captured end-systolic and end-diastolic stages of the cardiac cycle. Next, we processed the images and calculated various metrics using our custom image processing and analysis software. The custom software was developed in Matlab (The Mathworks, Natick, USA) Version 2012B. The application allows the user to load a high-bit depth image series in PNG format, sort, and select images of systole or diastole, segment the outline of the ventricle and store results as comma-separated value files. The software is available open-source^1^.

### Matlab Interface

The graphical user interface allows the user to browse through the acquired images and select the frames corresponding to the maximal end-systole and end-diastole (Supplemental Figure 1C—Right Panel). Then on the selected images the ventricle is manually segmented (Supplemental Figure 1C—white dots). Using an Otsu-based thresholding function in MATLAB, we classified pixels into those that contain hemoglobin and those that do not. Finally, we quantified the number of hemoglobin-containing pixels (referred as Hb blush) within a manually segmented ventricle at the end of systole and diastole (Supplemental Figure 1D).

### Measurements

Based on hemoglobin pixel counts and the total ventricular area delineated by manual segmentation, we estimated the total ventricular end-diastolic area (*tEDA*), the total ventricular end-systolic area (*tESA*), percent change in the total ventricular area (Δ_%_*tA* = 100^*^(*tEDA*-*tESA*)/*tEDA*), end-diastolic blood area (*EDBA*), end-systolic blood area (*ESBA*), the stroke area (*SA* = *EDBA*-*ESBA*), and ejection fraction (*EF* = 100^*^SA/*EDBA*). We also derived a “myocardial fraction” metric where we estimated the myocardial area and referred to that as the myocardial mass index (MMI). To derive MMI, we assumed that during peak systole, where the myocardium is at its maximal contraction, most of the blood is ejected minimizing this confounding volume. We then subtracted the end-systolic blood area from the total ventricular end-systolic area assuming that the remainder of the segmented area would be a rough estimate of the myocardial mass at maximal contraction.

### Dye Injection

Tadpole raised and positioned in 1% agar as described above. Using microinjector (Harvard Apparatus PLI-100 Pico-Injector Microinjection System) Brilliant Blue FCF (Sigma-Aldrich) dye injected to the cardinal vein. Movie obtained with Redlake/IDT Y4 Lite grayscale camera.

### Statistics

For comparison we utilized the Mann-Whitney test (non-parametric and not paired) and used a scatter plot with the bar showing the median and 95% confidence interval. We also included before-after graph including standard error of the mean. All significant differences between groups indicated in the figure legends. Significance was determined when the *p*-value is lower than 0.05.

## Results

### HCSA Imaging Can Detect Cardiac Dysfunction in the Setting of Sarcomere Perturbation

**Figure 1:**
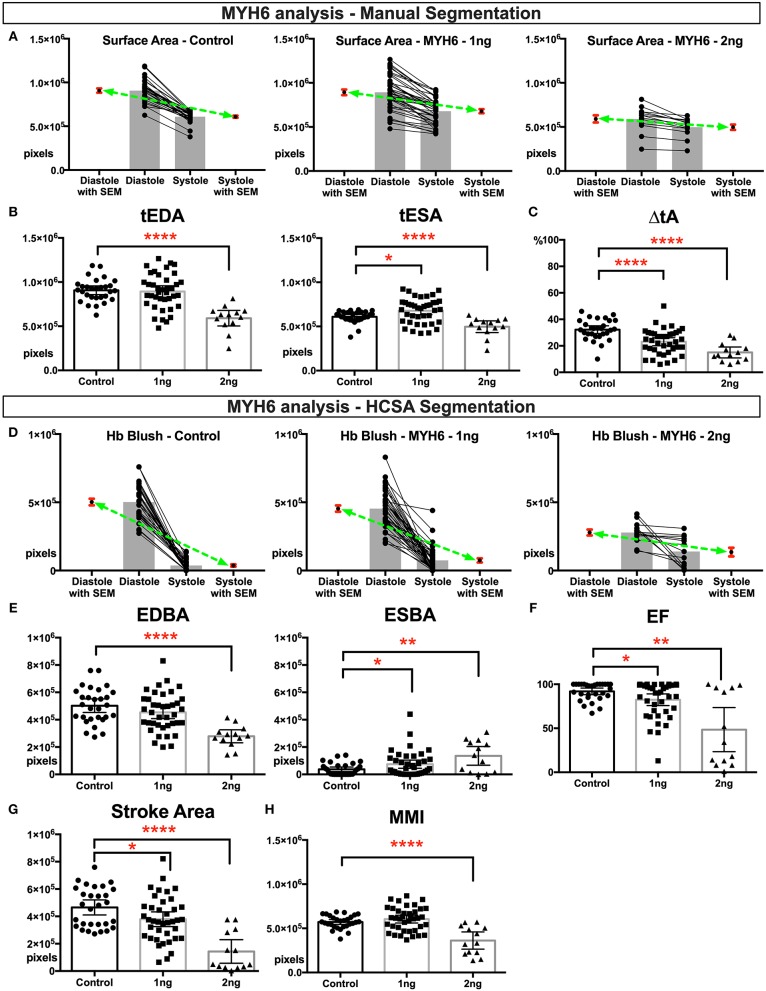
Physiological quantification of embryo heart function in MYH6 morphants. For this experiment 29 control, 39 morphants-−1 ng morpholino injected and 13 morphants-−2 ng morpholino injected analyzed. Measurements derived from manual segmentation include surface area at the end of diastole and systole and the change in ventricle area. **(A)** Before-after graph presented with extra columns pointing mean with SEM in red. Green line between means flattens as change in surface area diminishes with increasing MO dose. **(B,C)** As the MO dose is increased, the change in ventricle area is reduced, and the ventricle sizes are smaller. **(D)** Measurements derived from HCSA application include Hb blush at the end of diastole and systole and the change in blush area. Before-after graph presented with extra columns pointing mean with SEM in red. Green line between means flattens as change in blush area diminishes with increasing MO dose. **(E,F)** Following HCSA application, Hb blush is quantified at the end of diastole and systole. Ejection Fraction is derived from these measurments. Morphants demonstrated a dose dependent diminished ejection fraction and **(G)** stroke area. **(H)** Myocardial mass index estimates the amount of cardiac mass within the manually segmented heart. Myocardial mass index is affected in 2 ng morphants but remains within normal limits in 1 ng morphants. SEM, standard error of the mean; Hb Blush, Hemoglobin-containing pixels; tEDA, total end-diastolic area; tESA, total end-systolic area; ΔtA, change in total area; EDBA, end diastolic blood area; ESBA, end-systolic blood area; EF, ejection fraction; MMI, myocardiac mass index; HCSA, Hb contrast subtraction angiography. ^****^p < 0.0001, ^**^p < 0.01, and ^*^p < 0.05.

We first analyzed cardiac function in MYH6 morphant group. We analyzed HCSA images from 29 control embryos, 39 embryos injected with 1 ng MYH6 morpholino, and 13 embryos injected with 2 ng MYH6 morpholino. Overall, the 2 ng-morphant hearts were smaller in size as ascertained using total ventricular end-diastolic area (*tEDA*) (Figures 1A,B), whereas 1 ng-morphants had similar end diastolic area but slightly larger end systolic area when compared to the controls. The percent change in the ventricle area during contraction (Δ_%_*tA*) was significantly reduced in both morphants suggesting myocardial dysfunction (Figure 1C). We derived these measurements with manual segmentation as described in methods. Then we applied HCSA, where we were able to quantify end-diastolic and end-systolic Hb blush (*EDBA*: end-diastolic blood area; *ESBA*: end-systolic blood area), which is a surrogate for blood area. In end-diastole, due to their overall smaller cardiac size, 2 ng morphants filled with less blood whereas 1 ng morphants, given their normal cardiac size, filled with a similar amount of blood when compared to the controls (Figures 1D,E). Importantly, at the end of systole, they both showed a significant failure in emptying the ventricle leading to an increase in end systolic blood area (Figures 1D,E). Therefore, the ejection fraction and the stroke area diminished, so we concluded that the magnitude of the dysfunction was worse in 2 ng morphants (Figures 1F,G). Then we estimated MMI at maximal ventricular contraction. As shown in Figure 1H, 1 ng *myh6* morphants did not have a significant reduction in myocardial mass in contrast to 2 ng morphants where MMI was significantly reduced. In conclusion, 2 ng morphants showed both structural loss of myocardium size and functional loss of contractility. Nevertheless, even without a change in MMI in 1 ng *myh6* morphants, HCSA imaging could still delineate changes in myocardial function.

**Figure 2:**
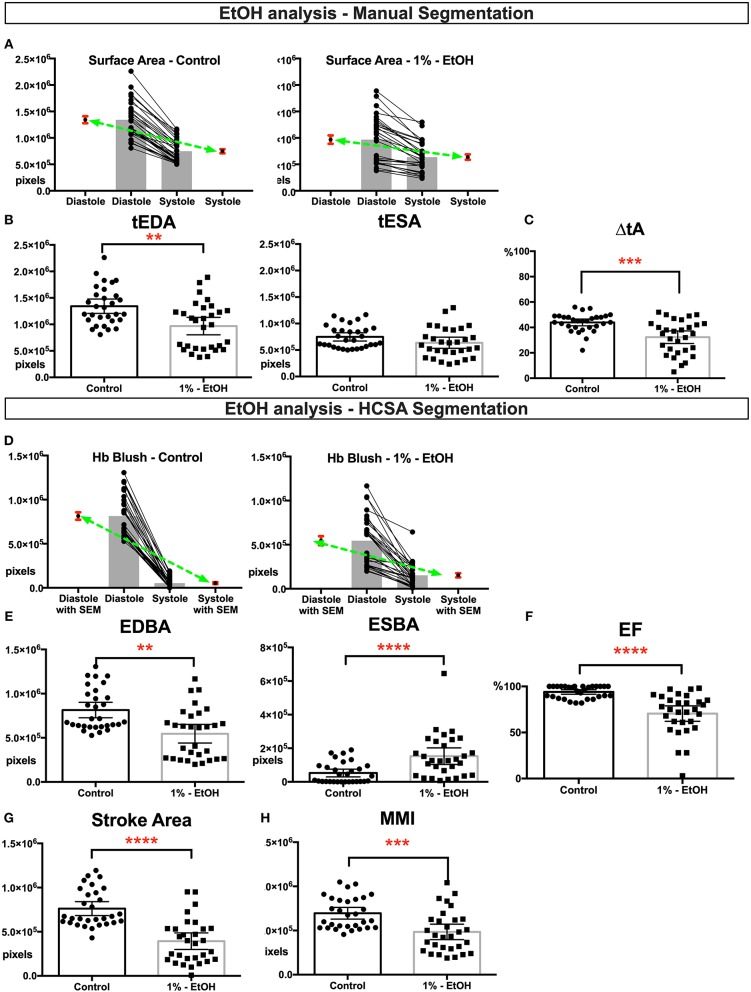
Physiological quantification of embryo heart function in EtOH treated tadpoles. For this experiment 30 controls and 30 EtOH exposed tadpoles analyzed. Measurements derived from manual segmentation includes surface area at the end of diastole and systole and the change in ventricle area. **(A)** Before-after graph presented with extra columns pointing mean with SEM in red. Green line between means flattens as change in surface area diminishes in EtOH group. **(B,C)** Ventricles of the ethanol exposed tadpoles were slightly smaller in size but had diminished change in the ventricle area. Following HCSA application, Hb blush quantified at the end of diastole and systole. Ejection fraction is derived from these measurements. **(D)** Before-after graph presented with extra columns pointing mean with SEM in red. Green line between means flattens as change in blush area diminishes in EtOH group. **(E,F)** Ethanol exposed tadpoles had less blood at the end of diastole and blood is not propelled efficiently leading an increase in end systolic Hb blush, diminished ejection fraction, and diminished **(G)** stroke area. **(H)** Myocardial Mass Index. In ethanol exposed tadpoles myocardial mass is diminished. SEM, standard error of the mean; Hb Blush, Hemoglobin-containing pixels; tEDA, total end-diastolic area; tESA, total end-systolic area; ΔtA, change in total area; EDBA, end diastolic blood area; ESBA, end-systolic blood area; EF, ejection fraction; MMI, myocardiac mass index; HCSA, Hb contrast subtraction angiography. ^****^p < 0.0001, ^***^p < 0.001, and ^**^p < 0.01.

Next we analyzed the ethanol exposed tadpoles. In the ethanol group, we analyzed HCSA images from 30 control and 30 exposed tadpoles. Grossly dysmorphic embryos were excluded. In this group, ventricles were smaller than the control group (Figures 2A,B) and the change in ventricle area was diminished (Figure 2C), suggesting that EtOH exposed ventricle can‘t shorten appropriately. Aslo *ESBA*, a surrogate for blood volume at the end of systole, was significantly higher than controls (Figures 2D,E) which is also likely due to the reduced ability to generate adequate ventricular force, shown as reduced ejection fraction and stroke area (Figures 2F,G). Therefore, together these tadpoles had slightly smaller looking ventricles with reduced myocardial pump function. Furthermore, “myocardial fraction” in systole shows a significant reduction suggesting a loss of myocardial mass (Figure 2H).

## Discussion

The incidence of CHD is staggering: ~1% of live births and 10% of aborted fetuses have CHD worldwide (Bernier et al., 2010; van der Linde et al., 2011; Jorgensen et al., 2014). New genomics technologies are enabling genetic analyses of CHD patients and identifying sequence variations in patients with CHD (Fakhro et al., 2011; Zaidi et al., 2013; Glessner et al., 2014; Homsy et al., 2015; McKean et al., 2016; Priest et al., 2016; Jin et al., 2017; Hoang et al., 2018; Manheimer et al., 2018). Genes identified in genomic studies are many but their role in cardiac development and function is uncertain; therefore, functional assays must be relatively rapid and inexpensive. We have previously shown that *Xenopus tropicalis* with the CRISPR/Cas9 system enables the analysis of hundreds of different genes in cardiac development (Deniz et al., 2018) and in this brief report we demonstrate that HSCA imaging can be used to screen for types of CHDs that present with sarcomeric dysfunction, for example cardiomyopathies.

The advantage of our HCSA method is that we can quantitate different metrics across multiple embryos to identify statistically significant differences between groups rather than relying on a qualitative assessment of function. In *myh6* morphants with increasing doses of morpholino we showed worsening cardiac function, yet 1 ng morphants didn't show differences in EDA, EDBA, and MMI suggesting that even subtle changes in sarcomeric function can be demonstrated with HCSA imaging. Similarly EtOH treated hearts were smaller overall and failed to eject adequate blood, again suggesting sarcomeric dysfunction that was easily quantified with HCSA imaging.

As a major advantage HCSA imaging is non-destructive, reproducible, faster, and most importantly non-lethal to the tadpole enabling multiple viewing session at various stages. This is important in analyzing numerous candidate genes. Fluoroscopy-based angiography requires microinjection in tadpoles, is fatal preventing subsequent imaging, and is difficult to reproduce limiting its utility for screening purposes (Movie 1). As the high-speed color cameras (>250 fps) are more available and cheaper, more detailed functional analysis including simultaneous assessment for cardiac and peripheral vasculature structures will be more feasible (Movie 2) providing structural details comparable to angiography images.

Clinical cardiology has demonstrated that imaging modalities (e.g., echocardiography and cardiac angiography) are essential to identify abnormal heart structures and heart function (Cua and Feltes, 2012; Roest and de Roos, 2012). Current advances in optical imaging are enabling comprehensive, high-resolution cardiac imaging in small animal models, which facilitates studying gene-phenotype and teratogen-phenotype relationships (Choma et al., 2010)*. Xenopus* is ideal for these studies given the ease of *in vivo* optical access to the cardiac structures as well as the optimal balance between human modeling and cost/efficiency (Warkman and Krieg, 2007). In this report we showed the effects of a human cardiomyopathy gene (MYH6) and a known teratogen, EtOH (known to cause Fetal Alcohol Syndrome) on early cardiac development using HCSA imaging. Broadly our results support the utility of HCSA imaging, when paired with *Xenopus*, to screen the impact of novel candidate CHD genes or potential teratogens on cardiac function.

## Data Availability Statement

All datasets generated for this study are included in the manuscript/Supplementary Files.

## Ethics Statement

Xenopus tropicalis were housed and cared for in our aquatics facility according to established protocols that were approved by the Yale Institutional Animal Care and Use Committee (IACUC) in accordance with NIH guidelines. Female and male mature Xenopus tropicalis were obtained from National Xenopus Resource.

## Author Contributions

ED, SJ, MK, and MC designed the study. ED and SJ acquired and analyzed the data. ED wrote the first draft of the article. SJ, MK, and MC contributed to the interpretation of the results and the writing of the manuscript. All authors have approved the final manuscript.

### Conflict of Interest

The authors declare that the research was conducted in the absence of any commercial or financial relationships that could be construed as a potential conflict of interest.

## Footnotes

^1^http://mhealthgroup.net/software (Note to editors and reviewers: the software will be made available upon publication of the manuscript).

## Supplementary Material

Supplemental Figure 1 Experimental and quantitative analysis workflow. **(A)** For *myh6* experiments, one cell *Xenopus* embryos are injected with either MOs and the tracer dye (Alexa488) or only the tracer dye (control group). In ethanol exposure experiments, we incubated post-midblastula stage embryos in1% EtOH. On day five, NF Stage 44 tadpoles are embedded in low melt agarose (1%) for non-pharmacological immobilization to facilitate imaging. **(B)** Scout Image and Magnified Images acquired. **(C)** Custom software interface. Right panel allows the user navigate through the acquired images and adjust the brightness and the contrast. Once maximal end-diastolic and maximal-end-systolic frames are identified, the ventricle is manually segmented. **(D)** Images processed and Hb blush is quantified within the segmented area using the HCSA software interface. IVF, *in vitro* fertilization; lta, left truncus arteriosus; rta, right truncus arteriosus; v, ventricle; oft, out flow tract; Hb, hemoglobin.

Movie 1 Grayscale movie demonstrating injection of Brilliant Blue dye via cardinal tail vein and tracking of flow from the venous system sequentially to the atrium, ventricle, and outflow tract that eventually propagates to the systemic circulation.

Movie 2 High speed (250 fps—color) color movie and processed HCSA movie.
