# Supplementary figures and images for: Quantitative Phenotyping of Xenopus Embryonic Heart Pathophysiology Using Hemoglobin Contrast Subtraction Angiography to Screen Human Cardiomyopathies
Source: Front Physiol. 2019 Sep 20;10:1197. doi: 10.3389/fphys.2019.01197 (PMC6763566; doi:10.3389/fphys.2019.01197)

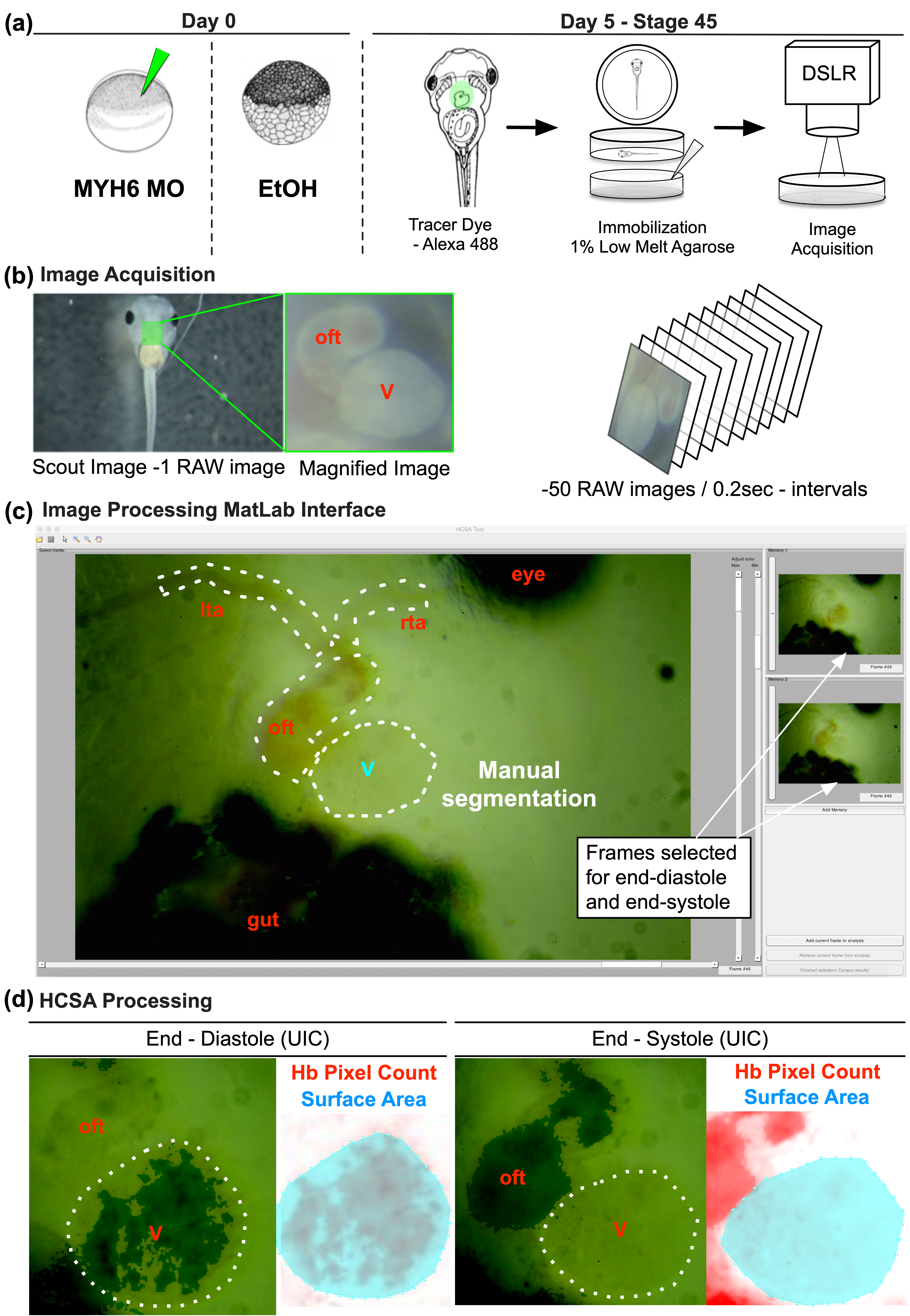

Supplement: Supplemental Figure 1 — Experimental and quantitative analysis workflow. (A) For myh6 experiments, one cell Xenopus embryos are injected with either MOs and the tracer dye (Alexa488) or only the tracer dye (control group). In ethanol exposure experiments, we incubated post-midblastula stage embryos in1% EtOH. On day five, NF Stage 44 tadpoles are embedded in low melt agarose (1%) for non-pharmacological immobilization to facilitate imaging. (B) Scout Image and Magnified Images acquired. (C) Custom software interface. Right panel allows the user navigate through the acquired images and adjust the brightness and the contrast. Once maximal end-diastolic and maximal-end-systolic frames are identified, the ventricle is manually segmented. (D) Images processed and Hb blush is quantified within the segmented area using the HCSA software interface. IVF, in vitro fertilization; lta, left truncus arteriosus; rta, right truncus arteriosus; v, ventricle; oft, out flow tract; Hb, hemoglobin. [file Image_1.JPEG]
